# Microstructure and Mechanical–Tribological Properties of HVOF-Sprayed (WC-Co+Ni) Coatings on Ductile Cast Iron

**DOI:** 10.3390/ma19122640

**Published:** 2026-06-18

**Authors:** Marzanna Ksiazek, Lukasz Boron, Adam Tchorz

**Affiliations:** 1 Department of Non-Ferrous Metals, AGH University of Krakow, al. A. Mickiewicza 30, 30-059 Krakow, Poland; 2 Lukasiewicz Research Network—Krakow Institute of Technology, 73 Zakopianska Str., 30-418 Krakow, Poland; lukasz.boron@kit.lukasiewicz.gov.pl (L.B.); adam.tchorz@kit.lukasiewicz.gov.pl (A.T.)

**Keywords:** HVOF, WC-Co coatings, Ni addition, microstructure, wear resistance, adhesion, tribology

## Abstract

High Velocity Oxy-Fuel (HVOF) thermal spraying enables the deposition of dense coatings with low porosity, high hardness, and good fracture resistance. Tungsten carbide–cobalt (WC-Co) coatings are widely used in industrial and aerospace applications due to their excellent wear resistance; however, improving crack resistance and coating–substrate adhesion remains a key challenge. In this study, WC-Co+Ni composite coatings were deposited on ductile cast iron, with emphasis on the role of Ni addition in controlling microstructure development under HVOF conditions. Microstructural characterization was performed using optical, scanning, and transmission electron microscopy (OM, SEM, TEM), while phase composition and chemical analysis were determined by X-ray diffraction (XRD) and energy-dispersive spectroscopy (EDS). The coatings exhibited a dense, low-porosity microstructure composed of fine WC and W_2_C carbides embedded in a Co–Ni binder, with locally nanocrystalline regions. XRD analysis confirmed WC and W_2_C as the dominant phases, with weak reflections corresponding to the η-phase (Co_6_W_6_C), indicating local decarburization. The addition of Ni increases the fraction of the transient liquid phase during particle flight, enhancing carbide dissolution and mass transport in the binder, which accelerates decarburization kinetics and promotes η-phase formation. Simultaneously, Ni modifies the binder into a more ductile Co–Ni matrix, reducing the detrimental effect of brittle η-phase on coating integrity. Mechanical and tribological testing (instrumented indentation and scratch testing) demonstrated improved crack resistance, wear resistance, and adhesion. The results show that Ni addition enables process-driven microstructural tailoring of HVOF-sprayed WC-Co coatings, leading to enhanced performance despite the presence of η-phase.

## 1. Introduction

Tungsten carbide–cobalt (WC-Co) composite coatings are widely used protective materials in surface engineering due to their excellent resistance to abrasive and erosive wear. Their performance results from the combination of hard WC particles embedded in a relatively ductile cobalt binder phase, which provides a balanced combination of hardness, fracture toughness, and load-bearing capacity [1,2]. WC-Co coatings exhibit superior abrasive and erosive wear resistance, which has been extensively documented in tribological studies [3,4]. Consequently, these coatings are applied in demanding industrial environments, including cutting and forming tools, casting dies, mining equipment, and mechanical components exposed to severe contact stresses and impact loading.

Among the available deposition techniques for carbide-based coatings, High Velocity Oxy-Fuel (HVOF) thermal spraying is widely used to produce dense and mechanically robust coatings [5,6,7]. In this process, powder particles are accelerated to supersonic velocities in a high-temperature combustion jet and impact the substrate in a semi-molten state [5,6]. Rapid splat deformation and solidification result in a lamellar microstructure with low porosity and strong intersplat cohesion [7,8]. Compared with conventional plasma spraying, HVOF deposition limits excessive carbide dissolution and decarburization while maintaining high coating density and mechanical integrity [6,9]. The final properties of the coatings are strongly influenced by processing parameters such as particle velocity, temperature, spray distance, and carrier gas pressure, which govern microstructure, porosity, and residual stress [7,10].

During HVOF deposition, complex thermochemical reactions occur within the particle stream and during splat solidification, often resulting in partial decarburization of WC and the formation of secondary phases such as W_2_C and η-phase carbides (e.g., Co_6_W_6_C) [11,12,13,14,15]. These phenomena are strongly related to phase stability and microstructural evolution in HVOF-sprayed WC-based coatings [14,15]. The resulting phase constitution significantly influences coating hardness and fracture resistance [14,15]. While moderate phase transformation may enhance hardness and wear resistance, excessive formation of brittle phases can reduce fracture toughness and accelerate coating degradation under cyclic or impact loading [11,14,15]. Therefore, controlling phase evolution during thermal spraying remains a critical challenge in the development of high-performance WC-based coatings [14,15].

Recent studies have explored the modification of WC-Co feedstock powders through the incorporation of metallic alloying elements such as Ni, Cr, Al, and complex alloys to improve coating performance. Alloying primarily affects the metallic binder phase, increasing its ductility, facilitating stress relaxation during rapid solidification, and enhancing intersplat cohesion [16,17,18]. Moreover, suitable alloying strategies can suppress excessive WC decarburization, limit the formation of undesirable secondary phases, and promote a more homogeneous distribution of the binder phase within the lamellar structure [18,19]. These microstructural modifications can alter wear mechanisms, shifting the dominant response from brittle carbide pull-out toward matrix-controlled plastic deformation, thereby improving wear resistance and coating durability [17,19,20,21].

Nickel additions have received particular attention due to their beneficial influence on coating integrity and mechanical stability. Ni-containing matrices exhibit increased ductility and improved resistance to cracking under tribological loading, which reduces microcrack formation and carbide fragmentation [22,23,24], while similar improvements in crack resistance and wear behavior have been widely reported in HVOF-sprayed WC-based coatings. Similar improvements in microstructural refinement and hardness have been associated with TiC additions, which contribute to grain growth inhibition and enhanced mechanical properties in WC-Co-based systems [25]. Additionally, grain-growth inhibitors such as VC and Cr_3_C_2_ can suppress carbide coarsening and promote microstructural refinement, leading to improved mechanical stability and coating durability [26,27].

The microstructure of HVOF-sprayed WC-Co coatings is governed by rapid thermomechanical interactions involving partial particle melting, splat deformation, and solidification. These processes produce a lamellar structure composed of WC/W_2_C particles embedded in a metallic matrix [28]. The size, morphology, and spatial distribution of carbide particles play an important role in determining hardness, fracture toughness, and tribological behavior [28]. Furthermore, tribochemical reactions during sliding contact may form protective tribofilms that influence friction and wear resistance, directly linking microstructural features with operational performance [29].

Ductile cast iron is commonly used as a substrate material due to its favorable mechanical strength, good machinability, and thermal expansion compatibility with carbide-based coatings. Its relatively high ductility makes it particularly suitable for applications requiring a load-bearing substrate combined with a hard, wear-resistant surface layer, such as in power engineering components. In high-velocity thermal spraying processes such as HVOF, substrate ductility also contributes to improved stress accommodation at the coating–substrate interface, reducing the risk of interfacial cracking. Coating performance strongly depends on the integrity of this interface, including adhesion strength and residual stress distribution [16,17,18]. In this context, the ductile cast iron substrate can synergistically interact with the Ni-modified Co–Ni binder, further enhancing strain accommodation at the interface and supporting improved coating durability under mechanical loading.

In this context, the present study aims to modify the chemical composition of WC-12Co powders by incorporating nickel particles and to deposit the resulting coatings on ductile cast iron using the HVOF process. Particular attention is paid to the effect of this modification on the microstructure and the mechanical and tribological properties of the coatings.

## 2. Materials and Methods

### 2.1. Preparating of Coating

Coating: WC-Co+Ni was applied by supersonic flame spraying of carbide powder containing WC-12Co (88 wt.% WC-12 wt.% Co) of grain size −45 + 5 µm, (Diamalloy 2002 Salzer-Metco, Pfäffikon, Switzerland) onto a ductile iron substrate. The WC-Co+Ni composite coating was obtained by introducing 10% of 20 µm Ni particles into the carbide powder. The powder mixture used to produce the composite coating consisted of 79.2 wt.% WC, 10.8 wt.% Co and 10.0 wt.% Ni. The HV-50 HVOF System supersonic spraying system at Plasma System S.A. (Siemianowice Śląskie, Poland) was used for spraying the coatings, in which a mixture of aviation kerosene and oxygen was used as fuel for the spraying process. Coating application parameters are listed in Table 1. Substrate made of EN-GJS-500-7 ductile iron with the following chemical composition: 3.61% C, 2.29% Si, 0.45% Mn, 0.045% P, 0.009% S, 0.03% Cr, 0.01% Ni, 0.057% Mg, 0.75% Cu, and the rest Fe, (in weight percentage), and was characterised by the following mechanical properties: Y.S = 340 (MPa) T.S = 500 (MPa), Elongation = 7%, Hardness = 220 HB. The substrate samples had dimensions of 100 × 15 × 5 mm^3^. Before spraying, the surface of the substrates has been treated with a loose corundum of 20 mesh granulation to improve the mechanical adhesion of coatings. The substrate surface roughness parameter R_a_ was 30 μm. The average coating thickness was 280 µm.

### 2.2. Microstructure Characterization

**Figure 1:**
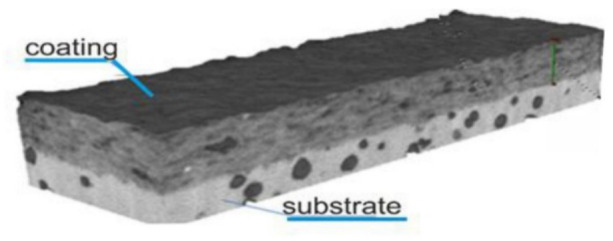
Cuboid-shaped coating/substrate sample (~2 mm) used for XCT analysis.

An optical microscope (MO) Axio Observer Zm1 by Zeiss (Jena, Germany), a scanning electron microscope (SEM) Dual Beam Scios FEI (FEI Company, Hillsboro, OR, USA), and a transmission microscope (TEM, JOEL 2010 ARD) (JEOL Ltd., Tokyo, Japan) equipped with EDS spectrometers were used to study the microstructure and chemical composition of the coating/substrate system. Preparations of the coating/substrate type for the transmission microscope in the form of a thin film were obtained by using ion thinning in a special device, the Gatan PIPS691V3.1 (Pleaasanton, CA, USA) for low-angle thinning [30]. Phase composition studies were carried out on the X’Pert Pro Panalytical Diffractometer (Malvern Panalytical Ltd., Almelo, Netherlands) in the angular range of 20–90° with CuK radiation. After such measurements, the obtained spectra were subjected to preliminary numerical processing using the “EVA” software (Bruker AXS GmbH, Karlsruhe, Germany, version 13.0), consisting of background subtraction and noise reduction using the Fourier transform. Phase identification was carried out with the help of the ICDD PDF-4+ (International Centre for Diffraction Data, Newtown Square, PA, USA, accessed 22 April 2026). Based on Rietveld analysis of XRD data using GSAS/EXPGUI software (General Structure Analysis System, Los Alamos National Laboratory, Los Alamos, NM, USA; EXPGUI interface version 2.0, GSAS version II), a set of phase compositions was derived. The average crystallite size was calculated from the Scherrer formula after taking into account the instrumental broadening. The porosity of the carbide coating was evaluated using X-ray computed tomography (XCT) on a Phoenix Nanotom nanotomograph (GE Sensing & Inspection Technologies, Wunstorf, Germany) with AxioVision image analysis software (AxioVision, Carl Zeiss Microscopy GmbH, Jena, Germany, version 4.8. Measurements were conducted on 10 regions of the coating using cuboid-shaped coating/substrate samples of approximately 2 mm in size (Figure 1).

Studies of the surface topography of the coatings and the determination of the surface roughness parameters R_a_ (average roughness value) and R_z_ (average roughness height) were carried out using a LEXT OLS4100 laser confocal microscope from OLYMPUS (Tokyo, Japan). Three measurement lines of coating surface roughness were used to calculate the parameters for each type of coating. Three-dimensional images and their analysis allowed for precise recognition of the geometric structure of the tested surfaces.

### 2.3. Mechanical and Tribological Properties

**Figure 2:**
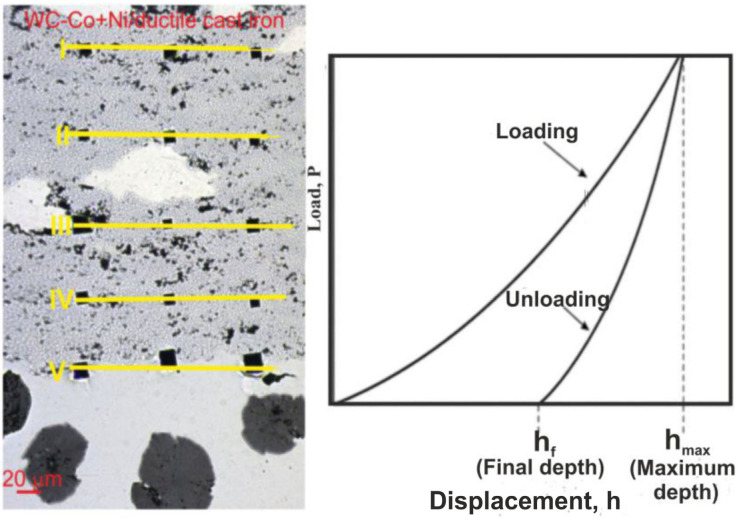
Measurement of microhardness (H_IT_) by matrix distribution on the cross-section of the coating and typical relationship between load and displacement during indentation.

Studies of mechanical properties, which included indentation measurements of hardness (H_IT_), Young’s modulus of elasticity (E_IT_), and fracture toughness (K_IC_), were carried out on the multifunctional measurement platform Micro Combi Tester of Swiss Company CSM Instruments (Peseux, Switzerland). H_IT_, E_IT_, and K_IC_ were determined by sample indentation (cross-section of coating/substrate samples) using a Vickers diamond indenter. Measurements continuously recorded the load and penetration depth during the loading and unloading cycles. The maximum load value for the hardness measurement and the Young’s module was 1 N, the load and unload speed was 2 N/min, the maximum load maintenance time was 10 s, and the contact force was 0.03 N. For The analysis of micromechanical properties was based on the Oliver and Pharr method, according to which the hardness (H_IT_) and Young’s modulus of elasticity (E_IT_) were calculated from the penetration curve (Figure 2). The measurement of the microhardness was done by a matrix distribution consisting of 15 measuring points on the cross-section of the coating for each coating/substrate system (Figure 2). The measurement positions along one measuring line: I, II, II, IV, and V were precisely defined with the special “Visual Advanced Matrix” module thanks to the integrated light microscope.

**Figure 3:**
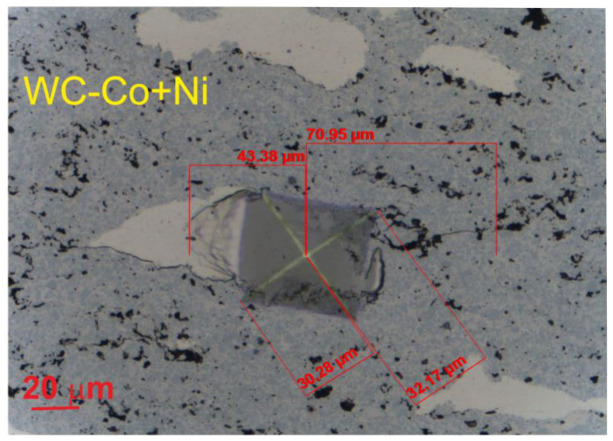
Scheme for measuring indentation fracture toughness (K_IC_) in the WC-Co+Ni coating.

Indentation fracture toughness was determined by the K_IC_ parameter, i.e., the critical value of the stress intensity coefficient, by direct measurement of the length of cracks that appeared in the corners as a result of the penetration of a Vickers indenter under the influence of a given load: 5, 10, 15, and 20 N (the speed of loading and unloading was 40 N/min, maximum load holding time was 10 s, and contact load was 0.03 N). For this purpose, the lengths of the cracks and the lengths of the indentation diagonals were determined using an integrated light microscope (Figure 3). Three indentations were made in each coating/substrate type sample at a given load. After determining the total length of the cracks, the type of cracks was identified, taking into account the length ratio l/a. When the l/a ratio is >1.5, the Anstis formula [31] is used. To determine the fracture toughness, take into account two parameters: the load (P) and the length of the crack (l).

Anstis formula:

(1) $$K_{\mathrm{IC}}\;=\;0.016\cdot {\left(\;\frac{E}{HV}\;\right)}^{0.5}\;\cdot \;\frac{P}{c^{1.5}}$$

where K_IC_—fracture toughness coefficient, *P*—indenter load [N], *HV*—Vickers hardness, *E*—Young’s modulus of elasticity [MPa], *c* = a + l—length of half of the indent’s diagonal + length of the crack initiated from corner of Vickers indent [m], a—length of half of the indent’s diagonal [µm], l—length of the crack initiated from corner of Vickers indent [µm].

The strength of the coating/substrate joint was evaluated using a four-point bending test performed on an INSTRON 8800M testing machine (Instron Corporation, Norwood, MA, USA). A specially designed fixture was used for specimens with dimensions of 36 × 13 × 3 mm^3^. The support span was 25 mm, and the crosshead displacement rate was set to 1 mm/min. For each test condition, three specimens were examined. After the bending tests, fracture surfaces were analyzed using scanning electron microscopy (SEM).

The bending strength was calculated according to the formula:

(2) $$\sigma =\frac{3}{2}\cdot F_{f}\frac{l}{d\cdot h^{2}}$$

where *σ*—bending strength [MPa] *F_f_*—destructive force [N], *l*—load spacing [mm], *d*—width of the specimen [mm], *h*—height of the specimen [mm].

**Figure 4:**
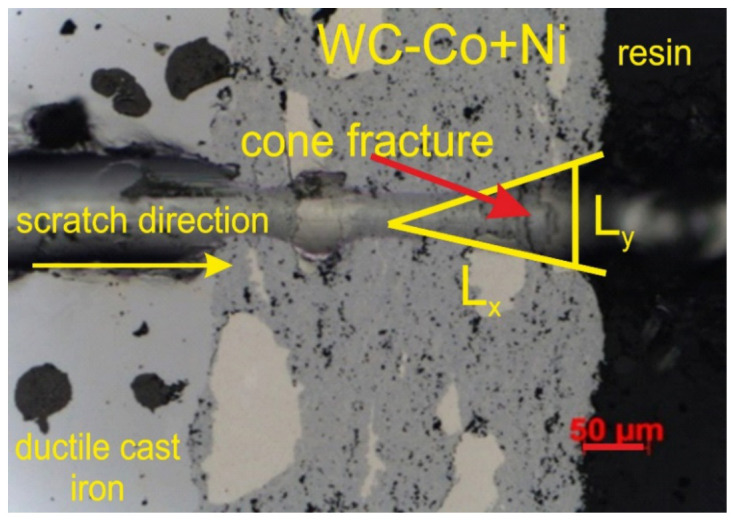
An example image of a scratch track in the substrate/coating/resin system. (F = 10 N).

Tests of adhesion of coatings to the substrate and determination of other mechanical types of damage, such as depth of penetration of the indenter, cracks, and the beginning of delamination in the crack profile of the scratch path, were carried out using a scratch test using a Rockwell C-type diamond indenter with a radius of curvature of 100 µm with a penetrator force of 5, 10, 15, 20, and 25 N, using a multifunction measuring platform (Micro-Combi Tester, Peseux, Switzerland) equipped with Anton Paar scratch test heads according to the standard [32]. The tests were carried out on the cross-sectioned samples embedded in Durofast hard epoxy resin and then polished using standard metallographic procedures. The scratch test was performed under a constant load, with the indenter moving from the substrate through the coating into the resin in which the sample was embedded. The scratch lengths were 1.2 mm and 2.4 mm, and the indenter speed was 0.4 mm/min. Failure of the coating/substrate system was detected and evaluated by examining the resulting scratch using light microscopy (LM) and scanning electron microscopy (SEM). Furthermore, the projected area of the cone-shaped fracture within the coating was determined after the scratch test as A_cn_ = L_x_ × L_y_ (Figure 4). For the constant load condition, this parameter was used to assess coating cohesion and wear resistance, based on measurements performed using a light microscope.

The abrasive wear resistance of ductile cast iron and the WC-Co+Ni/ductile cast iron coating system was evaluated using a ball-on-disc tribometer (Elbit Innovation and Implementation Company, Koszyce Małe, Poland) at ambient temperature. Tests were conducted for 1000 s under a normal load of 25 N and a rotational speed of 3500 rpm. The tribological pair consisted of a stationary sample plate and an Al_2_O_3_ ball (radius 0.6 mm, diameter 1.2 mm) as the counterbody, moving along a circular track with a radius of 5 mm. The sample was pressed against the ball with a constant, precisely controlled normal force, while the counterbody was mounted on a rigid fixture to ensure stable contact. Friction force, sliding distance, rotational speed, contact temperature, wear depth, and wear rate were continuously recorded using dedicated software. Linear wear of the sample was measured with a high-precision displacement sensor. Post-test analyses of wear track morphology and elemental composition were performed to identify dominant wear mechanisms and the nature of tribological interactions.

## 3. Results and Discussion

### 3.1. Microstructure and Phase Composition of the (WC-Co+Ni)/Ductile Cast Iron System

**Figure 5:**
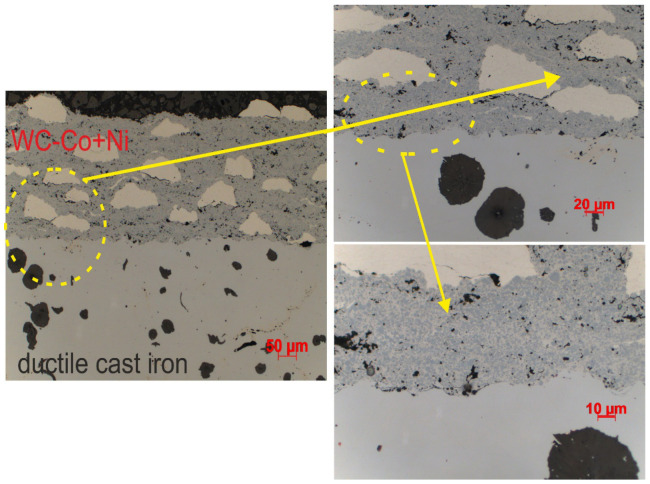
Microstructure of the (WC-Co+Ni/) ductile cast iron system at low and high magnification.

The microstructure of the composite (WC-Co+Ni) coating deposited on ductile cast iron, observed by optical microscopy (Figure 5), exhibits a typical lamellar morphology characteristic of HVOF-sprayed coatings. It consists of flattened splats formed as a result of particle impact and severe plastic deformation upon collision with the substrate. Fine WC particles are relatively uniformly distributed within the cobalt-based binder matrix, contributing to the structural homogeneity of the coating. The microstructure also reveals the presence of larger, highly deformed metallic regions corresponding to nickel particles, which underwent partial or complete melting during the spraying process (Figure 5). These Ni-rich regions form continuous metallic bridges between lamellae, enhancing interlamellar cohesion and improving overall ductility. As commonly observed in thermally sprayed composite systems, the low-melting metallic phase exhibits a higher degree of plastic deformation and effectively fills interlamellar voids between carbide particles. As a result, the coating exhibits a dense and compact structure with a low level of defects. It is characterized by low, isolated porosity, quantified as 3.2 ± 0.6% based on tomographic analysis, as well as a limited presence of oxides, mainly in the form of thin layers at lamellar boundaries. The coating–substrate interface is continuous and well developed, with no evidence of substrate melting, indicating that mechanical interlocking is the dominant adhesion mechanism in the HVOF process. The addition of Ni plays a key role in microstructural development by enhancing particle plasticization and promoting pore filling, thereby improving interlamellar cohesion and reducing structural defects. Consequently, the coating exhibits a high degree of densification, strong adhesion to the substrate, and a well-balanced distribution of hard ceramic and metallic phases. The presence of the ductile Ni phase contributes to enhanced stress accommodation, improved crack resistance, and increased overall coating toughness, which is particularly beneficial under mechanical loading conditions typical for tribological applications.

**Figure 6:**
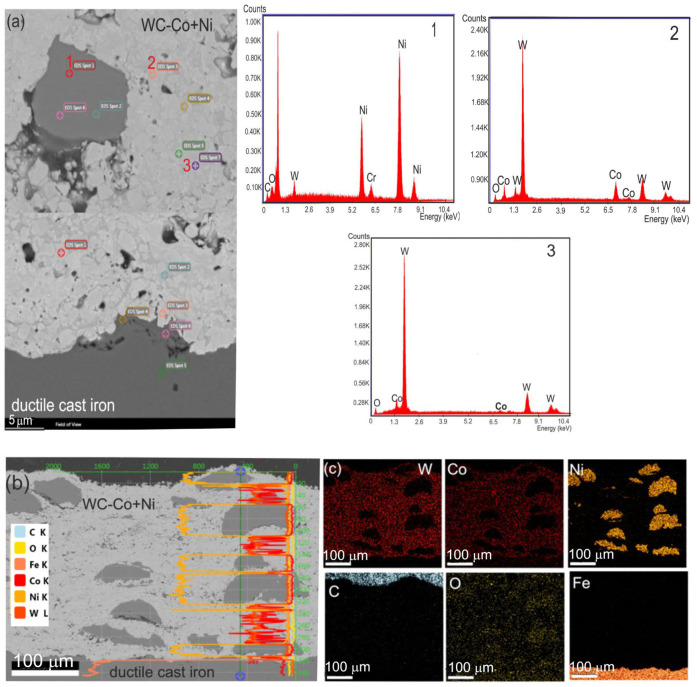
(**a**) Cross-sectional SEM images of the composite (WC-Co+Ni) coating with EDS spectra taken from the marked points 1,2, and 3: (**b**) linear representation of concentrations of C, O, Fe, Co, Ni, and W; (**c**) mapping the distribution of W, Co, Ni, C, O, Fe taken from the region of interface.

**Figure 7:**
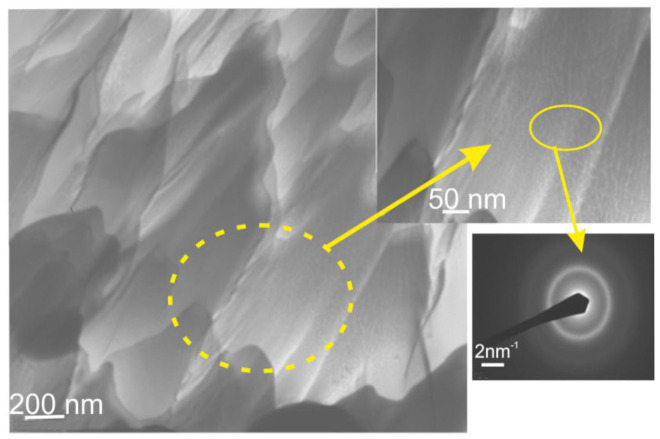
TEM image of the composite (WC-Co+Ni) coating with a representative selected area diffraction pattern, indicating the formation of a highly refined microstructure. The selected area electron diffraction (SAED) pattern reveals a highly defected, nanocrystalline structure with a significant quasi-amorphous component.

Observations performed using scanning electron microscopy (SEM), supported by energy-dispersive X-ray spectroscopy (EDS), revealed significant refinement of tungsten carbide particles from an initial size of approximately 40 µm to 0.5–2.5 µm after the spraying process (Figure 6). This refinement was observed both within the coating bulk and in the vicinity of the coating–substrate interface. EDS analysis confirmed the presence of elements characteristic of both the coating and the substrate, while elemental mapping revealed distinct concentration gradients across the interface. Transmission electron microscopy (TEM) investigations of thin regions of the (WC-Co+Ni) coating revealed a nanocrystalline microstructure with a banded morphology (Figure 7). The observed parallel bands, with thicknesses in the range of approximately 100–300 nm, indicate pronounced phase refinement and preferential alignment along the spray direction. Such a microstructure is conducive to increased hardness and improved tribological performance, while maintaining a high degree of interfacial cohesion.

**Figure 8:**
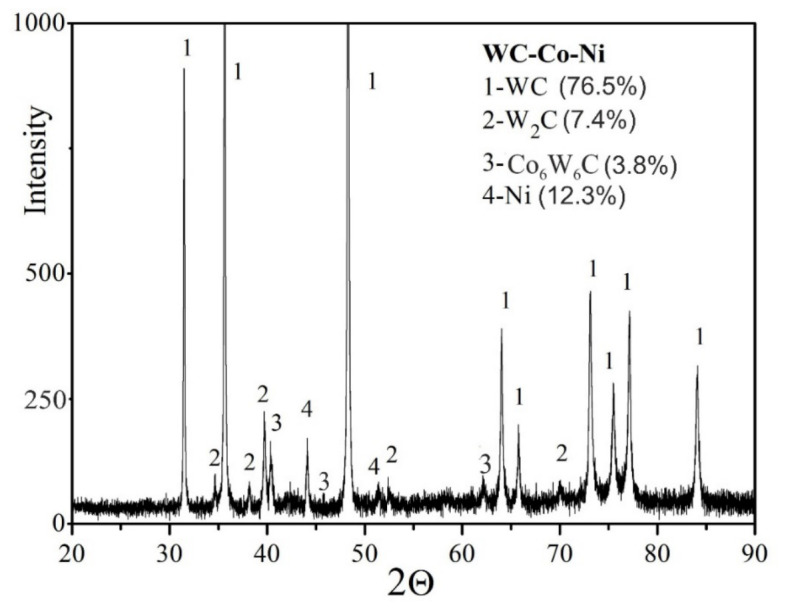
XRD pattern of the composite (WC-Co+Ni) coating.

X-ray diffraction (XRD) analysis of the WC-12Co coating modified with 10 wt.% Ni indicates that WC is the dominant crystalline phase, as evidenced by the highest intensity of its characteristic diffraction peaks (Figure 8). Semi-quantitative analysis suggests a phase fraction of approximately 76.5%, although this value should be considered as an estimate due to the limitations of XRD-based quantification. In addition to WC, the presence of W_2_C (7.4%) is identified, indicating partial decarburization of WC during the HVOF spraying process. Furthermore, weak reflections corresponding to the η-type phase Co_6_W_6_C (3.8%) are observed. The formation of this phase is typically associated with carbon deficiency and interaction between tungsten and the metallic binder under high-temperature conditions. No distinct diffraction peaks corresponding to metallic Co are detected. This may be attributed to its partial consumption during η-phase formation, as well as possible dissolution within the Ni-containing binder phase. The presence of Ni (12.3%) is confirmed by characteristic reflections, suggesting that Ni remains at least partially in a separate crystalline form or contributes to a Co–Ni solid solution, which may not be easily distinguishable from pure Co due to peak overlap. Overall, the identified phase composition reflects partial transformation of the initial feedstock during spraying, with decarburization and binder-phase modification playing a key role in the evolution of the coating microstructure. Such phase evolution is consistent with previously reported behavior of WC-based coatings subjected to decarburization during thermal spraying [12,14,15]. This is particularly important from the perspective of functional properties, as WC is characterized by very high hardness (HV ≈ 20–24 GPa), which governs its excellent resistance to abrasive wear, while exhibiting relatively low fracture toughness (K_IC_ ≈ 3–5 MPa·m^1/2^). The presence of the W_2_C phase (7.4%) indicates partial decarburization of WC during spraying. However, its content below 10% suggests that this process was controlled and did not lead to significant deterioration of coating properties. It should be noted that W_2_C exhibits lower fracture toughness (K_IC_ ≈ 2–4 MPa·m^1/2^), and higher fractions may promote microcrack initiation. The presence of the η phase (Co_6_W_6_C, 3.8%) confirms the occurrence of local diffusion reactions between the carbide phase and the metallic matrix. This phase, recognized as brittle (K_IC_ ≈ 1.5–3 MPa·m^1/2^), may reduce fracture resistance; however, its low fraction indicates that these transformations were limited and did not significantly affect the mechanical performance of the coating. The Ni phase content, at approximately 12% (slightly above the nominal 10 wt.%), can be attributed to changes in phase equilibrium during spraying, including partial carbon loss. Nickel, similarly to cobalt, acts as a ductile phase, enhancing the matrix’s ability to accommodate and relax residual stresses. The metallic matrix exhibits relatively high fracture toughness (on the order of 40–100 MPa·m^1/2^), which contributes to inhibiting crack propagation initiated in brittle carbide phases. In the context of these results, it should be emphasized that the formation of decarburization products (W_2_C and η phase) is inevitable in high-temperature processes; however, their limited fraction is of key importance. Additionally, the fine-grained and locally nanostructured morphology of the carbide phases may further promote crack deflection and energy dissipation. In summary, the obtained phase composition (WC—76.5%, W_2_C—7.4%, Co_6_W_6_C—3.8%, Ni—12.3%) indicates a well-developed microstructure in which the hard WC phase dominates, with a moderate contribution of decarburization products. This configuration provides an effective balance between high hardness and resistance to brittle fracture, characteristic of high-quality (WC-Co+Ni) coatings deposited by the HVOF method.

**Figure 9:**
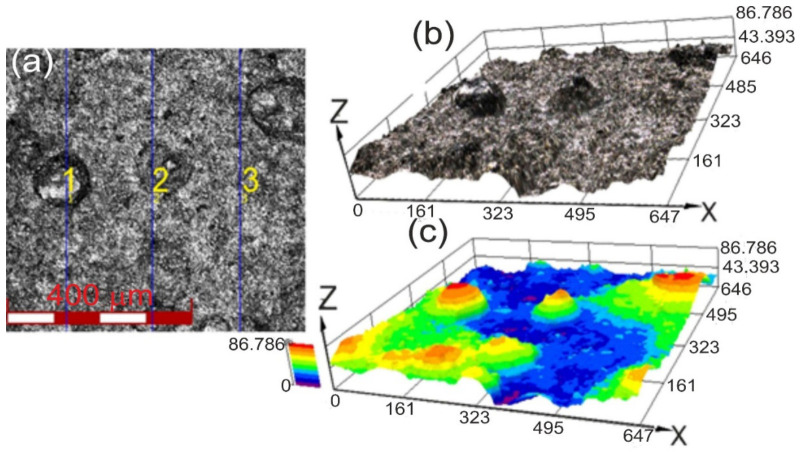
Surface morphology of the composite (WC-Co–Ni) coating on a cast iron substrate observed using a confocal microscope (200× magnification): (**a**) 2D intensity image, (**b**) 3D height reconstruction, (**c**) 3D color reconstruction.

Figure 9 shows the measurement location of surface roughness of the composite (WC-Co+Ni) coating, recorded using a laser confocal microscope at 200× magnification, together with a three-dimensional reconstruction of the surface topography. Analysis of the obtained images enabled a detailed assessment of the surface geometrical structure. The roughness parameters of the investigated coating were determined as R_a_ = 3.87 ± 0.14 µm and R_z_ = 14.40 ± 1.56 µm. The relatively high surface roughness is mainly associated with the composite nature of the coating and the presence of nickel particles, which appear as elongated, partially flattened features with an irregular, island-like distribution within the matrix. The presence of asperities of varying sizes contributes to increased surface development. An increase in the fraction and size of Ni particles leads to higher roughness, which should be considered when interpreting scratch test results, particularly in terms of adhesion and wear mechanisms. At the same time, such surface topography may enhance wear resistance through improved mechanical interlocking.

### 3.2. Mechanical Characteristics of (WC-Co+N)i/Ductile Cast Iron System

The micromechanical properties, including indentation hardness (H_IT_) and indentation Young’s modulus (E_IT_), determined on cross-sections of the (WC-Co+Ni)/ductile cast iron coating system, revealed significant variation with depth. The results summarized in Table 2 show that the average H_IT_ and E_IT_ values in the upper part of the coating are 10.05 ± 5.38 GPa and 244.78 ± 47.31 GPa, respectively. These values result from the coexistence of hard carbide phases and a ductile Ni-containing metallic phase within the microstructure. The observed variability is directly related to the heterogeneous lamellar structure of the coating and its phase composition (WC—76.5%, W_2_C—7.4%, Co_6_W_6_C—3.8%, Ni—12.3%). The presence of Ni increases the fraction of the ductile phase, promoting a more uniform stress distribution and enhancing the ability to accommodate deformation, thereby stabilizing the mechanical response. The highest hardness and Young’s modulus values were recorded in the central region of the coating (~100 µm from the substrate), which can be attributed to strain hardening during particle impact, improved interlamellar cohesion, and significant refinement of WC grains. The average H_IT_ values range from 9.39 to 14.23 GPa, with a maximum of 14.23 ± 3.85 GPa observed in regions enriched in hard phases. Locally elevated hardness values (up to ~18 GPa) correspond to indentations within WC particles, whereas lower values (~6–8 GPa) are characteristic of Co/Ni-rich regions. The variation in Young’s modulus (E_IT_ ≈ 241–291 GPa) confirms the structural heterogeneity typical of HVOF-sprayed coatings. Secondary phases such as W_2_C and Co_6_W_6_C locally increase stiffness and hardness, but may also enhance susceptibility to brittle deformation. In contrast, Ni-rich regions exhibit reduced hardness, lower stress concentration, and enhanced stress relaxation. The distribution of properties as a function of distance from the substrate indicates a general increase in hardness toward the coating surface. However, the absence of a clear monotonic trend suggests that the HVOF process ensured a relatively uniform phase distribution at the macroscopic scale, while local variations arise primarily from the lamellar structure and heterogeneous phase distribution. The largest scatter in H_IT_ values, observed in the coating–substrate interface region (±5.85 GPa), is attributed to residual stresses, local material mixing, diffusion effects, and partial decarburization and oxidation. The micromechanical response of the coating is governed by the interaction between hard carbide phases (WC, W_2_C, Co_6_W_6_C) and a Co-based metallic matrix modified by Ni, which is present both in dissolved form and as partially unmolten ductile particles. The dominant WC fraction ensures high load-bearing capacity, while Ni stabilizes the mechanical response, reduces stress concentration, and limits local damage, which is critical for service performance. The H/E ratio, commonly used as an indicator of elastic strain resistance and contact deformation stability, reflects the load-bearing capacity and sliding stability of the WC-Co+Ni coating [33]. The highest H/E value (0.049) was obtained in line III, indicating enhanced resistance to crack initiation, whereas Ni-rich regions exhibited lower values, promoting stress redistribution and strain accommodation (Table 3). Similarly, the H^3^/E^2^ parameter, representing resistance to plastic deformation, reached its maximum in line III (0.034 GPa), consistent with the increased fraction of hard carbide phases.

Fracture toughness (K_IC_) results of the (WC-Co+Ni) coatings, presented in Table 4 together with representative indentation imprints, were evaluated based on indentation-induced crack lengths and impression diagonals using established indentation fracture mechanics models. The calculations were performed using the measured hardness (H_IT_) and elastic modulus (E_IT_) values. The results indicate that both hardness and Young’s modulus play a significant role in governing fracture resistance. The highest K_IC_ values were recorded in matrix-dominated regions of the coating, whereas locally reduced values were observed in Ni-enriched areas. The presence of Ni, acting as a ductile phase relative to brittle WC carbides, promotes plastic deformation and suppresses crack propagation. As a result, the (WC-Co+Ni) coatings combine high hardness with enhanced plastic deformability, leading to improved resistance to wear and cracking. Higher K_IC_ values were obtained at lower loads (10–15 N), which is attributed to shorter crack lengths and a favorable E/H ratio that promotes plastic accommodation. The increased scatter of K_IC_ values in Ni-rich regions reflects the heterogeneous microstructure and local variations in mechanical properties. Furthermore, HVOF spraying has been reported to induce partial decarburization of WC particles and the formation of refined or locally nanocrystalline matrix regions, which may contribute to improved fracture resistance. Overall, the combined analysis of K_IC_, H_IT_, and E_IT_ confirms that local reductions in hardness in Ni-enriched regions enhance plastic deformability and suppress crack initiation, resulting in improved resistance to wear and fracture under tribological loading conditions.

**Figure 10:**
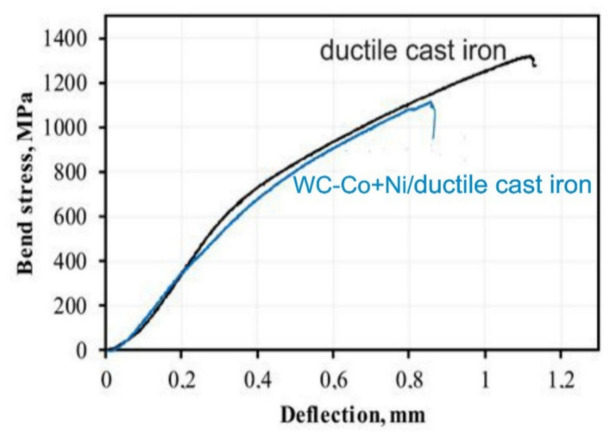
Bend test curves recorded for (WC-Co+Ni)/ductile cast iron system and ductile cast iron.

Figure 10 shows the results of the four-point bending test for the WC-Co+Ni/ductile cast iron system, presented as the relationship between bending stress and deflection. The maximum bending stress for the WC-Co+Ni coating reached 1097 MPa at a deflection of 0.86 mm, whereas for the uncoated ductile cast iron the corresponding values were 1272 MPa and 1.12 mm, respectively. Despite the slightly lower deflection, the coated system maintains a high load-bearing capacity under bending, indicating efficient stress transfer and redistribution at the coating–substrate interface. The presence of a fine-grained metallic matrix and partially melted, ductile Ni particles leads to a local reduction in the effective Young’s modulus and promotes stress relaxation. Under bending conditions, these Ni-rich regions act as local plastic deformation zones, reducing stress concentration at phase boundaries and limiting microcrack initiation.

**Figure 11:**
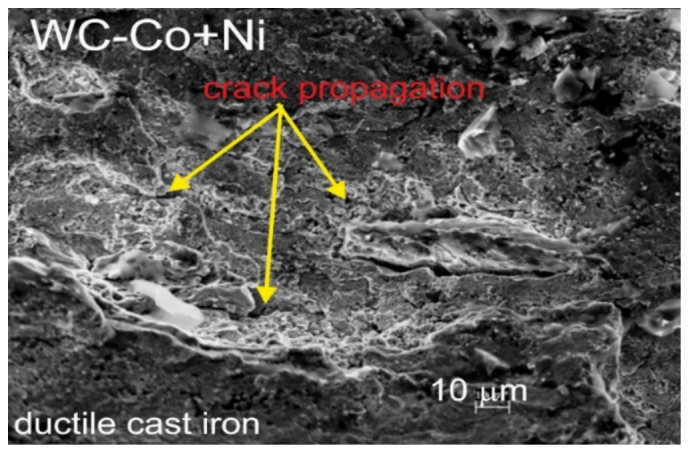
SEM micrograph of the fracture surface of the (WC-Co+Ni)/ductile cast iron system after bend test.

At the same time, they promote crack-bridging mechanisms, increasing the energy required for crack propagation. As a result, the coating exhibits high resistance to failure under bending conditions. Fractographic analysis after bending (Figure 11) confirms these observations. Cracks are observed to develop primarily within the coating and locally in the vicinity of the coating–substrate interface, without propagating into the ductile cast iron substrate. The presence of partially molten Ni particles acts as a local mechanical buffer, hindering crack growth and increasing resistance to crack propagation toward the substrate. This mechanism promotes effective dissipation of fracture energy within the coating and reduces stress transmission to the substrate. Consequently, the coating not only efficiently carries the applied load but also protects the substrate from excessive stress concentration, which is consistent with the observed high integrity of the coating–substrate interface. No signs of interfacial delamination were detected. In the analyzed system, differences in the coefficients of thermal expansion between the constituent phases and the substrate play a key role in the residual stress state. WC-Co-based coatings are characterized by a relatively low coefficient of thermal expansion (≈5–6 × 10^−6^ K^−1^), significantly lower than that of the iron-based substrate (~13.2 × 10^−6^ K^−1^). This mismatch promotes the development of compressive residual stresses in the coating during cooling, which may enhance resistance to crack initiation but requires partial stress accommodation to maintain adhesion.

The introduction of Ni particles, with a coefficient of thermal expansion close to that of the substrate (~13 × 10^−6^ K^−1^), partially compensates for this mismatch, reducing thermal strain gradients and limiting stress concentration within the coating microstructure. As a result, the WC-Co+Ni/ductile cast iron system exhibits high structural integrity, with no evidence of interfacial delamination, indicating that the residual stress level remains below the critical threshold for coating failure. The relatively similar elastic modulus values of the coating (E_IT_ ≈ 240–290 GPa) and the substrate (E ≈ 165 GPa for ductile cast iron) contribute to a reduced elastic mismatch, which may alleviate stress concentration in the interfacial region and promote more efficient load transfer across the coating–substrate interface. Fracture observations after bending tests reveal a mixed failure mode, consisting of cohesive fracture within the coating and localized interfacial cracking, confirming good interfacial adhesion and the absence of a dominant delamination mechanism. These observations are consistent with the analysis of the H/E and H^3^/E^2^ ratios, which indicate a balanced combination of hardness and elastic strain accommodation capability. In particular, the H/E ratio is associated with resistance to elastic deformation and crack initiation, whereas the H^3^/E^2^ parameter is related to resistance to plastic deformation. Together, these parameters suggest improved damage tolerance under mechanical and tribological loading conditions [33].

### 3.3. Tribological Characteristics of (WC-Co+Ni)/Ductile Cast Iron System

**Figure 12:**
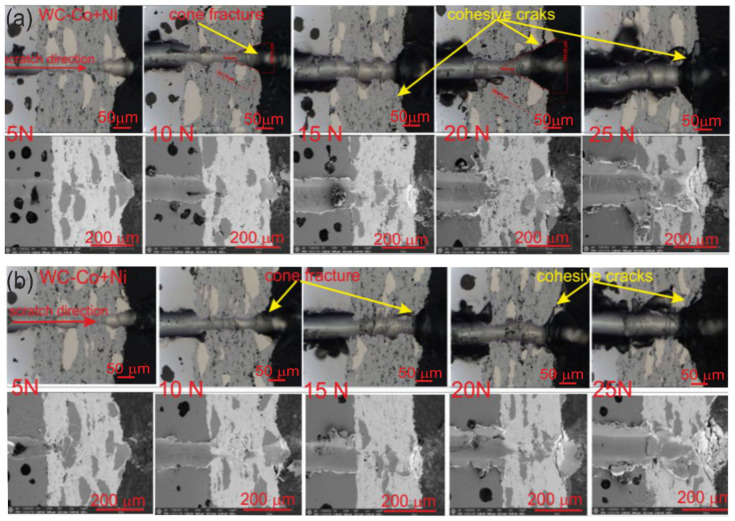
LM/SEM micrographs of damage evolution during the scratch bond strength test of the (WC-Co+Ni)/ductile cast iron system at increasing loads (5–25 N) for scratch lengths: (**a**) 1.2 mm and (**b**) 2.4 mm. The transition from initial damage to cone fracture, 15–20 N) and cohesive cracking, 25 N) is observed; scratch direction indicated.

Figure 12 shows the variation in the cone crack area (Acn) for two scratch lengths (1.2 mm and 2.4 mm) as a function of the applied load. At the lowest load (5 N), only the onset of damage is observed, without the formation of a fully developed cone crack. In both cases, Acn increases systematically with increasing load up to 20 N, reaching a maximum value of 37.74 × 10^−3^ mm^2^, indicating progressive cone crack propagation. This trend is confirmed by the micrographs in Figure 12, where well-developed cone fracture features are clearly visible in the load range of 15–20 N. Comparative analysis reveals that scratch length significantly influences the stability of cone crack evolution (Table 5). For the longer scratch length (2.4 mm), Acn exhibits a more regular, near-monotonic increase, suggesting a more stable stress distribution and more uniform crack propagation. In contrast, the shorter scratch length (1.2 mm) shows greater variability in Acn, which may be associated with localized stress fluctuations and increased sensitivity to microstructural heterogeneity. At 25 N, a deviation from the increasing trend is observed for both configurations, manifested either as a reduction in Acn (1.2 mm) or a deviation from monotonic growth (2.4 mm), suggesting a limitation in cone crack growth and a transition in the dominant damage mechanism. Microstructural observations (Figure 12) support this interpretation, as more complex damage morphologies, including cohesive cracking within the coating, become evident at the highest load. This behavior can be attributed to additional microstructural mechanisms associated with the presence of Ni particles, which may act as barriers to crack propagation through crack deflection and crack bridging. These mechanisms promote local energy dissipation and contribute to stabilization of cone crack growth. This effect is more pronounced for the longer scratch length, where a larger deformation volume enables averaging of local microstructural interactions. Overall, the results indicate that scratch length influences not only the magnitude of Acn but also the kinetics and stability of cone crack propagation, while the presence of Ni modifies the damage evolution and failure mechanisms, as reflected in the observed fracture morphology in Figure 12.

**Figure 13:**
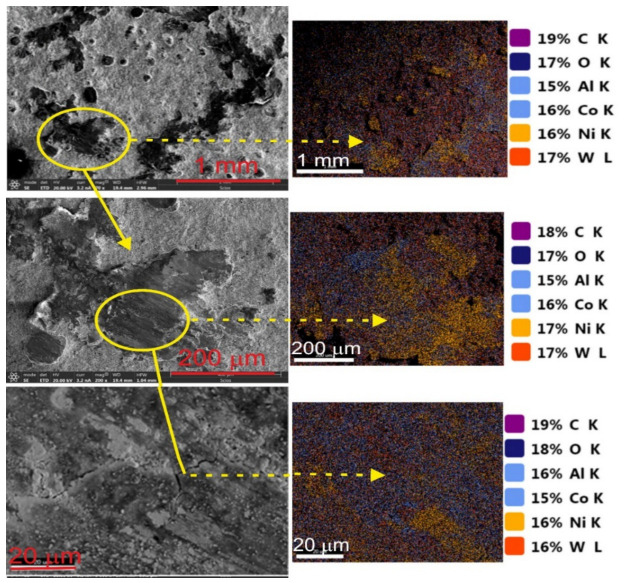
SEM images showing the wear morphology of the composite (WC-Co+Ni) coating along with the corresponding elemental maps.

**Figure 14:**
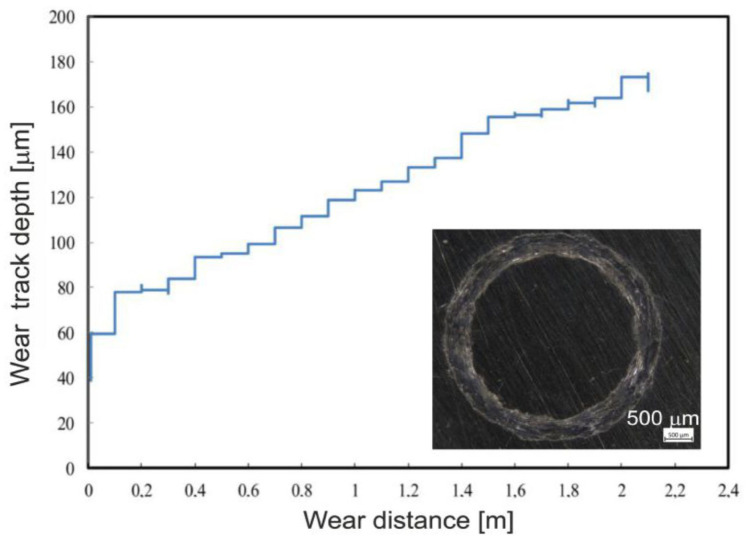
Dependence of the wear track depth of the WC-Co+Ni coating on the wear distance.

The morphology and composition of the wear track on the (WC-Co+Ni) coating were analyzed using SEM/EDS (Figure 13) together with the wear depth profile (Figure 14). The worn surface exhibits a heterogeneous morphology with grooves, pull-out craters, and compacted debris, indicating predominantly abrasive wear. EDS analysis reveals a non-uniform elemental distribution, where W- and C-rich regions correspond to WC/W_2_C fragments, while Co and Ni are associated with the metallic matrix. The presence of oxygen suggests tribo-oxidation, whereas aluminium originates from the Al_2_O_3_ counterbody, indicating material transfer. The wear depth profile shows a quasi-monotonic, step-like increase with wear distance, reflecting progressive material removal with local discontinuities related to carbide and carbide–matrix detachment. These fragments may act as loose abrasives, contributing to a three-body wear mechanism. The absence of abrupt depth increases indicates stable wear without dominant delamination. Overall, wear behavior is governed by microstructural heterogeneity and carbide–matrix cohesion, with Ni contributing to stabilization of the wear process by reducing stress concentration and limiting severe particle detachment.

## 4. Conclusions

From the conducted research and analysis of the results, the following conclusions can be drawn:

1. The (WC-Co+Ni) composite coating deposited by the HVOF technique exhibits a dense microstructure with low porosity and a well-developed, defect-free interface with the ductile cast iron substrate. The coating is characterized by a heterogeneous structure consisting of WC as the dominant phase embedded in a metallic Co–Ni binder. Additionally, microstructural refinement and locally nanocrystalline regions were identified (confirmed by TEM/SAED), which are attributed to rapid solidification and limited diffusion during the spraying process.
2. Phase analysis indicates that the WC-Co–Ni system should be considered as a pseudo-ternary (W–C–Co–Ni) system. The XRD results confirm the presence of WC and W_2_C phases, along with the formation of η-type phases (Co_6_W_6_C), while no distinct metallic Co phase was detected. This is attributed to decarburization processes during HVOF spraying and the consumption of Co in η-phase formation, as well as its partial dissolution in the Co–Ni binder.
3. The addition of Ni significantly affects the kinetics of phase transformations during spraying by promoting transient liquid phase formation, which enhances WC dissolution and contributes to local carbon depletion. As a result, Ni indirectly facilitates the formation of W_2_C and η phases, while simultaneously stabilizing a fine-grained Co–Ni binder that improves the overall microstructural integrity.
4. The presence of a refined and locally nanocrystalline Co–Ni matrix enhances mechanical performance through grain refinement strengthening (Hall–Petch effect), improved stress redistribution, and increased resistance to crack initiation and propagation. Although the formation of brittle η phases may locally reduce ductility, their negative effect is mitigated by the improved plasticity and cohesion of the modified binder.
5. The tribological behavior of the (WC-Co+Ni) coating results from the synergistic interaction between hard WC/W_2_C phases and the ductile Co–Ni matrix. While carbide phases provide high wear resistance, the modified binder improves load transfer, limits carbide pull-out, and promotes more stable and controlled wear mechanisms, leading to enhanced wear resistance under mechanical loading.
6. The obtained results demonstrate that (WC-Co+Ni) coatings deposited by HVOF on ductile cast iron substrates offer a balanced combination of high hardness, improved toughness, and enhanced wear resistance. This is primarily due to microstructural refinement, nanocrystalline features, and binder modification induced by Ni, despite the concurrent formation of secondary carbide and η phases under carbon-deficient conditions.

## Figures and Tables

**Table 1:** HVOF spraying parameters of as sprayed WC-Co+Ni coatings.

Gun Movement Speed (mm/s)	Oxygen (l/min)	Kerosene (l/h)	Powder Feed Rate (g/min)	Powder Feed Gas (l/min)	Spraying Distance (mm)
583	944	25.5	92	Nitrogen, 9.5	370

**Table 2:** Indentation hardness (H_IT_) and Young’s modulus (E_IT_) values of the (WC-Co+Ni)/ductile cast iron coating system.

Measuring Line	Region	H_IT_ [GPa]	E_IT_ [GPa]	Average H_IT_ [GPa]	Average E_IT_ [GPa]
I	Matrix (top)	13.28	288.83	10.05 ± 5.38	244.78 ± 47.31
		13.03	295.27		
		3.83	150.23		
II	Matrix (center)	11.57	266.13	9.39 ± 1.95	252.69 ± 12.51
		7.83	250.58		
		8.76	241.38		
III		10.52	238.69	14.23 ± 3.85	290.99 ± 47.85
		18.21	332.52		
		13.95	301.78		
IV	Matrix (bottom)	6.30	228.78	11.59 ± 4.59	268.09 ± 34.74
		14.01	294.60		
		14.46	280.93		
V	Interface	9.28	270.19	10.42 ± 5.85	269.31 ± 56.97
		16.76	325.85		
		5.22	211.88		

**Table 3:** Indentation hardness, Young’s modulus, and H/E and H^3^/E^2^ ratios of the composite (WC-Co+Ni) coating.

Measuring Line	Average H_IT_ [GPa]	Average E_IT_ [GPa]	H/E	H^3^/E^2^
I (top)	10.05	244.78	0.041	0.017
II (center)	9.36	252.69	0.037	0.013
III (center)	14.23	290.99	0.049	0.034
IV (bottom)	11.59	268.09	0.043	0.022
V (interface)	10.42	269.31	0.039	0.016

**Table 4:** Indentation fracture toughness measurements of the composite (WC-Co+Ni) coating under loads of 10, 15, 20, 25 and 30 N.

**Load**	H_IT_ = 4.92 GPa, E_IT_ = 189.92 GPa	H_IT_ = 12.18 GPa, E_IT_ = 250.03 GPa	H_IT_ = 5.32 GPa, E_IT_ = 198.97 GPa
**10 N**	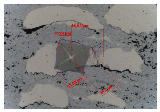 K_IC_ = 3.71 [MNm^−3/2^]	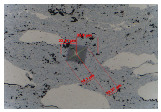 K_IC_ = 1.00 [MNm^−3/2^]	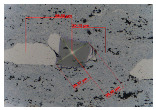 K_IC_ = 1.41 [MNm^−3/2^]
**15 N**	H_IT_ = 14.05 GPa, E_IT_ = 260.21 GPa 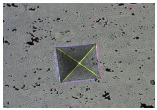 no crack	H_IT_ = 9.57 GPa, E_IT_ = 234.46 GPa 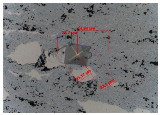 K_IC_ = 2.15 [MNm^−3/2^]	H_IT_ = 4.34 GPa, E_IT_ = 167.05 GPa 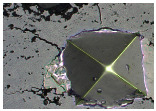 no crack
**20 N**	H_IT_ = 12.50 GPa, E_IT_ = 226.04 GPa 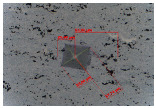 K_IC_ = 1.59 [MNm^−3/2^]	H_IT_ = 10.10 GPa, E_IT_ = 266.39 GPa 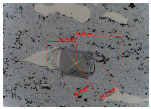 K_IC_ = 1.43 [MNm^−3/2^]	H_IT_ = 16.45 GPa, E_IT_ = 184.7 GPa 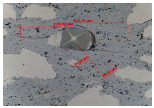 K_IC_ = 0.76 [MNm^−3/2^]
**25 N**	H_IT_ = 12.90 GPa, E_IT_ = 211.04 GPa 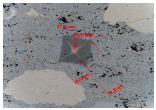 K_IC_ = 1.61 [MNm^−3/2^]	H_IT_ = 10.34 GPa, E_IT_ = 214.42 GPa 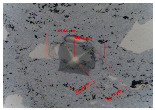 K_IC_ = 2.68 [MNm^−3/2^]	H_IT_ = 5.87 GPa, E_IT_ = 178.08 GPa 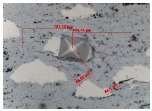 K_IC_ = 0.84 [MNm^−3/2^]
**30 N**	H_IT_ = 11.21 GPa, E_IT_ = 212.57 GPa 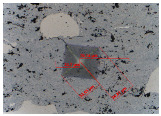 K_IC_ = 1.96 [MNm^−3/2^]	H_IT_ = 5.81 GPa, E_IT_ = 159.32 GPa 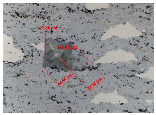 K_IC_ = 0.76 [MNm^−3/2^]	H_IT_ = 5.59 GPa, E_IT_ = 161.8 GPa 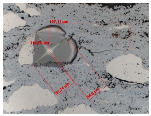 K_IC_ = 0.65 [MNm^−3/2^]

**Table 5:** Averaged scratch bond test results of the (WC-Co+Ni)/ductile cast iron system.

Scratch Length	Load [N]	Lx [µm]	Ly [µm]	A_cn_ × 10^−3^ [mm^2^]
1.2 mm	10	151.74	104.41	15.84
	15	108.86	152.68	16.62
	20	205.76	183.42	37.74
	25	115.51	259.56	29.98
2.4 mm	10	112.47	138.13	15.54
	15	127.50	201.98	25.64
	20	162.92	170.41	27.76
	25	170.71	198.34	33.86

## Data Availability

The original contributions presented in this study are included in the article. Further inquiries can be directed to the corresponding author.
